# No need to rethink sensorimotor circuits—Commentary on Goldblatt et al. (2024)

**DOI:** 10.3389/fneur.2025.1544855

**Published:** 2025-01-23

**Authors:** Joel C. Glover

**Affiliations:** Department of Molecular Medicine, University of Oslo, Oslo, Norway

**Keywords:** vestibulo-ocular, chicken, mouse, zebrafish, reflex, synaptic, specification, development

## Introduction

In the recently published article *Motor neurons are dispensable for the assembly of a sensorimotor circuit for gaze stabilization*, Goldblatt et al. (1) provide strong evidence that vestibulo-ocular projection neurons in the zebrafish acquire their functional identities in vestibulo-ocular reflex circuitry despite the absence of their motoneuron targets. This is touted as disproving a longstanding hypothesis, purported to have originated from me (2) and formalized by Straka (3), in which target extraocular motoneurons retrogradely specify vestibulo-ocular neuron identities. Goldblatt et al. (1) present this work as a major paradigm shift in how sensorimotor circuit development is conceived, a premise that is promoted by an accompanying commentary entitled *Neuronal development: Rethinking sensorimotor circuits* (4).

## Reviewing the history

**Figure 1:**
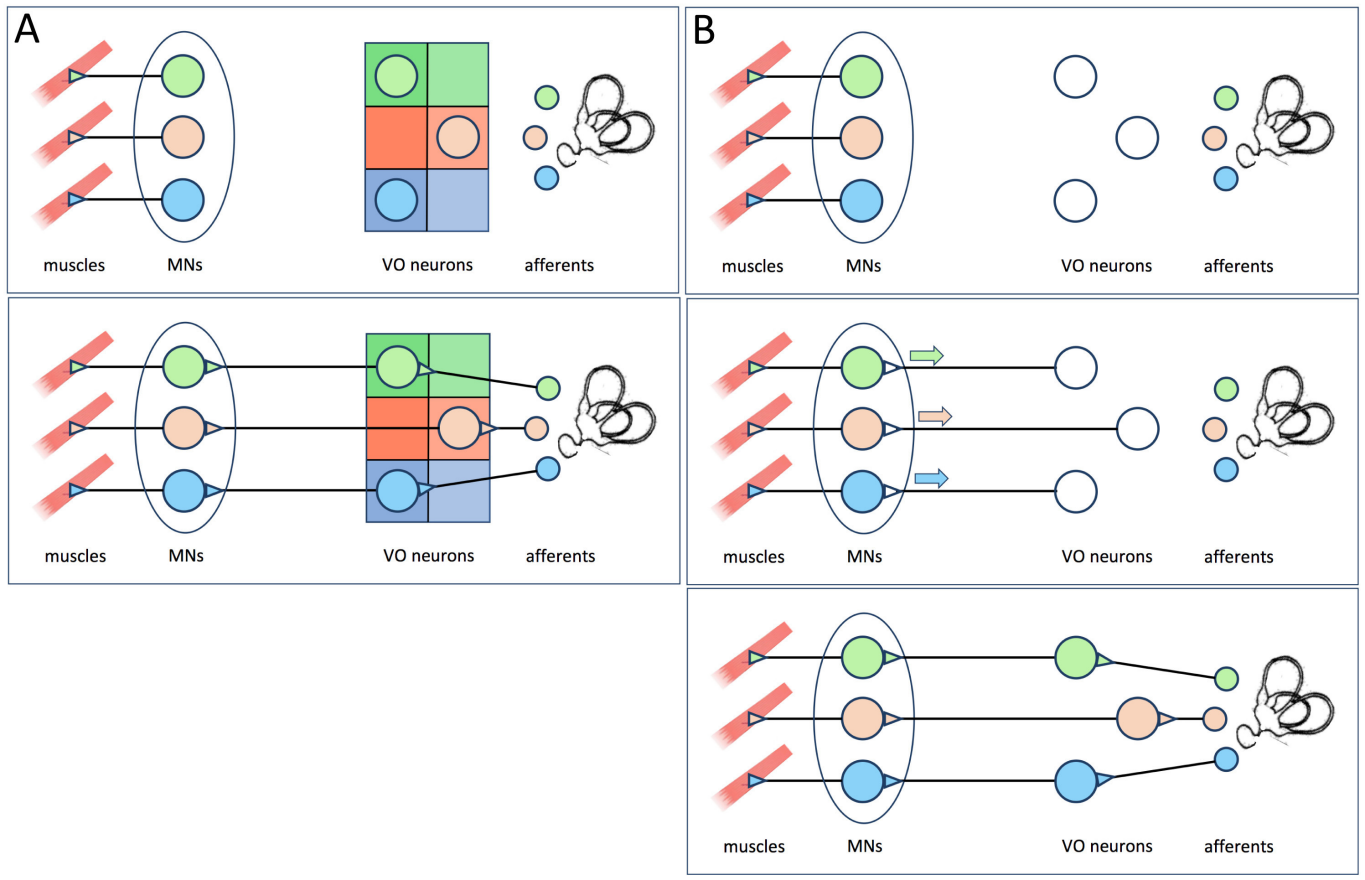
**(A)** The hypothesis proposed by Glover (5), reviewed in Glover (2) and Straka (3) as cited by Goldblatt et al. (1). The functional identities of vestibulo-ocular neurons within the vestibulo-ocular reflex circuit are specified by positional information imposed by rhombomeric and longitudinal domains within the hindbrain neuroepithelium (upper panel). This allows the vestibulo-ocular neurons to make appropriately selective connections with extraocular motoneurons and receive appropriately selective afferent inputs (lower panel). Note that according to this model, elimination of MNs would not prevent the formation of appropriately selective afferent inputs. **(B)** The hypothesis put forth by Goldblatt et al. (1), which they incorrectly attribute to Glover (2) and Straka (3). The functional identities of vestibulo-ocular neurons are initially unspecified (upper panel), but become so after contacting target MNs and receiving retrograde signals from them (middle panel). Once specified, the vestibulo-ocular neurons can then receive appropriately selective afferent input (lower panel).

There's just one problem with this narrative. It is simply not correct. I have never proposed the hypothesis that motoneurons retrogradely specify vestibulo-ocular neuron identities, and Hans Straka (now deceased) never formalized or promulgated it. Quite the contrary: the hypothesis that I proposed 3 decades ago (5) is that vestibulo-ocular projection neurons are specified according to their positions in the hindbrain neuroepithelium, through the actions of anteroposterior and dorsoventral expression domains of key transcription factors. They are not retrogradely specified after contacting their target motoneurons, but rather have already obtained their identities prior to projecting their axons to the motoneurons. Numerous publications from my laboratory since then have provided evidence to support the pre-specification of vestibulo-ocular neurons (see below). Thus, the results obtained by Goldblatt et al. (1), rather than “comprehensively overturning” it, support the working model of vestibulo-ocular reflex pathway development that originally sprang from research in my laboratory (Figure 1).

How then did Goldblatt et al. (1) and Zwart (4) manage to mix this up? To understand this, some historical background is in order.

In 1989, I presented an abstract at a meeting of the Society for Neuroscience demonstrating that vestibulospinal and vestibulo-ocular projection neurons with distinct axonal projection pathways are arranged in the early chicken embryo in coherent groups in a checkerboard-like pattern in register with the hindbrain rhombomeres and longitudinal domains intersecting these (6). Over the next few years, further investigation showed that these groups maintain their coherence through subsequent development (7), are synaptically linked to specific target motoneuron pools in the trochlear and oculomotor nuclei (8), that their lineages can be traced to specific rhombomeric and sub-rhombomeric domains (9), that the pattern arises before the vestibulo-ocular axons reach the motoneurons and the axons grow along specific pathways to the site of the target motoneurons unerringly (5, 10) and establish initially specific termination patterns (11), and that manipulations of activity known to disrupt topographic patterns of visual and somatosensory projections do not affect the connections from vestibulo-ocular axons to oculomotor motoneurons [reviewed in Glover (2)]. These findings led to the idea of the “vestibular hodological mosaic,” in which positional determinants specify the axon trajectories and functional identities of vestibular projection neurons [the idea was originally presented in Glover (5), and the term “hodological mosaic” was coined in Glover (12)]. Continued work in my laboratory and in collaboration with others demonstrated the same developmental organization of vestibular projection neurons in the mouse and used mouse transgenics to test the role of Hox genes (specifically HoxB1) in establishing it (13, 14). In parallel, Straka et al. (15) showed that the same organization pertains in larval frogs. More recently, the hypothesis that the distinct projection pathways and synaptic targets of vestibular projection neurons involves an early molecular specification was tested and supported by RNA sequencing of different vestibulospinal neuron groups (16). And in a recently submitted study (17) we show that vestibulo-ocular circuitry emerges with appropriate afferent selectivity as soon as the reflex arc is completed and despite all vestibulo-ocular neurons engaging in synchronous waves of activity, favoring some form of cellular recognition over patterned activity as the underlying substrate.

Over the years, these studies and their implications have been summarized in numerous review articles ((2, 3, 5, 12, 18–21, 24), and in an upcoming review will be placed in a broader hodological context (22). In all of these reviews, the following proposal is laid out clearly: vestibulo-ocular projection neurons are specified by their early positions, independently of and presaging their later selective synaptic contacts with motoneurons. Here from Glover (24):

“The correlation of cluster domains with unique fields of regulatory gene expression at early stages suggests a mechanistic link between the position of a vestibular interneuron^1^ and the differentiation of its axon trajectory, termination pattern, and neurotransmitter phenotype.”

## Correcting the misinterpretation in Goldblatt et al. (2024)

**Figure 2:**
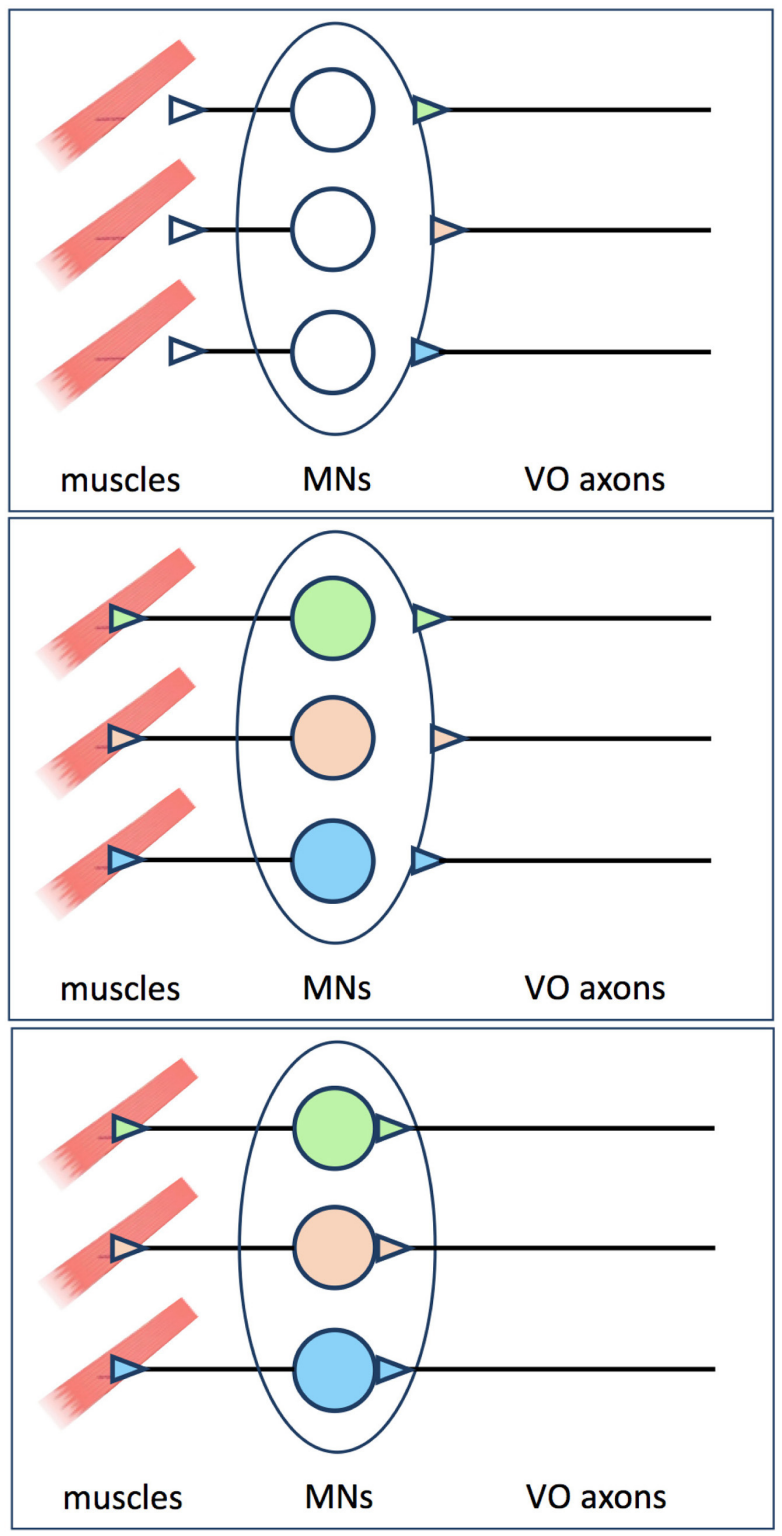
Proposal made by Glover (2) to explain why pre-specified vestibulo-ocular axons pause before innervating their target MNs. Axons from pre-specified vestibulo-ocular neurons reach the outskirts of the oculomotor and trochlear nuclei before MN identities are evident, and must therefore wait there before extending axon collaterals into the MN pools (upper panel). Retrograde signals from muscle are required before MN identities are specified or made available to presynaptic partners (middle panel). Once this occurs, the pre-specified vestibulo-ocular axons can proceed to extend collaterals and form synapses on their appropriate MN targets (lower panel).

Where then does the notion of retrograde specification from target motoneurons arise? Goldblatt et al. (1) and Zwart (4) appear to have latched on to a very specific (and interesting) feature of vestibulo-ocular reflex circuit development, originally documented in the chicken embryo. Once the axons of the already distinct vestibulo-ocular neuron groups have grown along their specific pathways to the site of their target motoneurons, they pause before extending axon collaterals into the individual motoneuron pools (2, 11). In the interim, the motoneurons make synaptic contacts with their target muscles. This led me to suggest that the motoneuron identities are not yet established, or not yet made available to the waiting presynaptic vestibulo-ocular axons, at the time the latter arrive on the scene. Thus, motoneuron identities may be retrogradely specified by contact with muscle or other features in the periphery [as appears to be the case for at least some motoneurons in the spinal cord, reviewed in Jessell et al. (23)], forcing the already specified vestibulo-ocular axons to wait patiently before they can consummate the predetermined synaptic relationship (Figure 2).

Thus, when Straka (3) in his review states that

“….the synaptic connectivity of the VOR pathway is established in reversed order to the signaling direction…” and “This suggests that VOR wiring is accomplished by a specification process that retrogradely transmits postsynaptic target identities to presynaptic neurons…,”

the *sequence in which synapses are made* is retrograde, but it is the *specification of motoneuron identities* and the transmission of these to the presynaptic vestibulo-ocular axons that is at play, not the specification of *vestibulo-ocular* neuron identities. Although the meaning of the second sentence might be misconstrued, uncertainty on this point can be quickly dispelled by a cursory perusal of the extensive literature cited above.

In their discussion, Goldblatt et al. (1) place considerable weight on the alternative notion that vestibular projection neuron identities arise through some sort of “intrinsic specification,” such as through the action of developmental patterning genes. What they fail to point out is that this is exactly the idea I proposed long ago.

## Discussion

Goldblatt et al. (1) present an excellent set of high-quality data. Their conclusion is clear: vestibulo-ocular neuron identities and upstream circuit connectivity do not depend on interactions with their postsynaptic motoneurons. But despite “comprehensively overturning” what they couch as “the strongest version of the retrograde specification model,” that model is still their invention, not mine or Hans Straka's. The “current” model—extant since I proposed it 30 years ago—states that the functional identities of vestibulo-ocular projection neurons (and likely all vestibular projection neurons) are specified long before their axons reach their motoneuron targets. This model remains intact. Indeed, it has been strengthened, not overturned, by Goldblatt et al. (1). There has thus been no paradigm shift, and no need to “rethink sensorimotor circuits,” at least not in the context of the vestibulo-ocular reflex.

## Footnotes

^1^Note that the term “interneuron” here is used generically for neurons interposed between sensory afferents and motoneurons.
